# Single and Combined Effects of *Clostridium butyricum* and *Saccharomyces cerevisiae* on Growth Indices, Intestinal Health, and Immunity of Broilers

**DOI:** 10.3390/ani8100184

**Published:** 2018-10-19

**Authors:** Mervat A. Abdel-Latif, Mohamed E. Abd El-Hack, Ayman A. Swelum, Islam M. Saadeldin, Ahmed R. Elbestawy, Ramadan S. Shewita, Hani A. Ba-Awadh, Abdullah N. Alowaimer, Hatem S. Abd El-Hamid

**Affiliations:** 1 Department of Nutrition and Veterinary Clinical Nutrition, Faculty of Veterinary Medicine, Damanhour University, Damanhour 22511, Egypt; mervat.abdellatif@vetmed.dmu.edu.eg; 2 Department of Poultry, Faculty of Agriculture, Zagazig University, Zagazig 44511, Egypt; m.ezzat@zu.edu.eg; 3 Department of Animal Production, College of Food and Agriculture Sciences, King Saud University, Riyadh 11451, Egypt; isaadeldin@ksu.edu.sa (I.M.S.); vet.hani77@gmail.com (H.A.B-A); aowaimer@ksu.edu.sa (A.N.A.); 4 Department of Theriogenology, Faculty of Veterinary Medicine, Zagazig University, Zagazig 44511, Egypt; 5 Department of Physiology, Faculty of Veterinary Medicine, Zagazig University, Zagazig 44511, Egypt; 6 Department of Poultry and Fish Diseases, Faculty of Veterinary Medicine, Damanhour University, Damanhour 22511, Egypt; Ahmed.elbestawy@vetmed.dmu.edu.eg (A.R.E.); drhatem_deltavet@yahoo.co.uk (H.S.A.E.-H.); 7 Department of Nutrition and Clinical Nutrition, Faculty of Veterinary Medicine, Alexandria University, Edfina 22758, Egypt; Ramadan_nutrition@yahoo.com

**Keywords:** probiotics, *C. butyricum*, *S. cerevisiae*, microbiota, performance, broilers

## Abstract

**Simple Summary:**

After banning the use of antibiotics as growth promoters in poultry production, scientists had to find efficient and safe alternatives. Because of their useful properties, two types of probiotic bacteria were chosen to be administrated in a broiler diet, either individually or combined. Useful effects were obtained regarding improvements of growth performance, and immunity.

**Abstract:**

A total of 120 1-day-old commercial Cobb chicks were used to study the effects of *Clostridium butyricum* (*C. butyricum*) and/or *Saccharomyces cerevisiae* (*S. cerevisiae*) on growth performance, intestinal health, and immune status in broilers. The experimental groups were as follows: G1; basal diet (BD), G2; basal diet (BD) plus *C. butyricum* preparation at 0.5 g/kg diet, G3; BD plus *S. cerevisiae* preparation at 0.5 g/kg diet, G4; BD plus 0.25 g/kg *C. butyricum* preparation plus 0.25 g/kg *S. cerevisiae*. Results showed that the total body weight gain, feed conversion efficiency, and protein efficiency ratio were significantly higher (*p* < 0.05) in the G4 group than in the other groups. The mortality percentage was reduced in the probiotic-supplemented groups. The villi height was elongated, and the villus height/crypt depth ratio was significantly increased in G2 and G4 chicks, compared to those in the control. The crypt depth was significantly decreased in all the probiotic-supplemented groups. Hemagglutination inhibition titers for Newcastle disease virus (NDV) were markedly increased in G2 and G4 chicks at 35 days of age, compared to those in G3 and control chicks. These results showed that dietary supplementation of a combined mixture of *C. butyricum* and *S. cerevisiae* in an equal ratio (G4) was more effective in improving growth performance, immune status, and gut health of broilers, compared with individual supplementation at a full dose.

## 1. Introduction

Antibiotics have been included in a poultry diet for many years to enhance growth rates, stabilize gut microbiota, and protect against some enteric diseases that cause significant loss in productivity. Due to the increase in the microbial resistance to antibiotics and residues in chicken meat products that are known to be harmful to consumers, the European Union (EU) has forbidden the use of antibiotic feed additives in poultry diets [1]. Therefore, there is a growing interest in finding alternatives (organic feeding) to antibiotics for poultry production [2]. Probiotics, prebiotics, and phytogenic feed additives with different ratios and combinations are important alternatives to antibiotics [3,4]. Moreover, lysozyme is considered as the most recent potentially effective feed additive that can be used to substitute for antibiotics [5].

It is well known that intestinal microbiota of chickens has a wide metabolic potential and it affects both the nutrition and health of the host. Increased counts of some harmful pathogens, such as *Clostridium perfringens* and *Escherichia coli*, may directly affect gut health, reduce nutrient absorption, bird growth, body weight gain (BWG), livability of birds and increase the feed conversion ratio (FCR). Therefore, it is considered as a strong indicator for poor intestinal integrity and digestion. This overgrowth of harmful bacteria might be related to decrease of beneficial bacteria as *Lactobacillus* counts.

Probiotics are live and harmless micro-organisms that assist in modulating intestinal microbiota for improving growth and utilization of feed in broiler chickens [6,7]. The use of probiotics in poultry should be based on their functions through the ability to competitively exclude pathogens from colonizing the intestine, which improves the gut flora and enhances the immunomodulatory activity, as well as the growth rate of the chicks. 

*Clostridium butyricum* (*C. butyricum*) is a gram-positive, anaerobic, spore-forming bacterium, isolated from soil, healthy animals, and human fecal matter, which produces butyric acid [8]. It can resist low pH and high bile concentrations. Moreover, *C. butyricum* can be used as an alternative to antibiotics as it can protect against infections [9]. Dietary supplementation of *C. butyricum* has been demonstrated by several researchers to promote growth performance [8,10], improve immune function [11,12], modulate nitrogen emissions, improve gut morphology, and maintain a balanced intestinal microflora in chickens [11,13].

*Saccharomyces cerevisiae* (*S. cerevisiae*) is one of the most widely distributed species of yeast used in animal nutrition. Upon addition of yeast to their diets at the end of the rearing period, the growth rate and feed utilization were enhanced in chicks [14,15], villus height in the ileum was increased [9], and through colonization of beneficial intestinal microflora, species was maintained through competitive exclusion [16,17]. Many studies have evaluated the effects of individual or a mixture of probiotic microbes of the same species. Chapman et al. [18] reported an increased response to multistrain, in comparison to monospecies probiotics which could be attributed to their synergistic interactions. A proper mixture of multimicrobe probiotics is an effective strategy to elicit the beneficial effect of *C. butyricum* and *S. cerevisiae*. Therefore, the present study aimed to evaluate the effect of *C. butyricum* or *S. cerevisiae*, individually and in combination, on growth, gut health, and immunity of broilers.

## 2. Materials and Methods

All procedures and experiments were performed in accordance with the ethics of the Committee of Local Experimental Animal Care and were approved by the Nutrition and Veterinary Clinical Nutrition Institutional Committee, Faculty of Veterinary Medicine, Damanhour University, Egypt (DMU2018-0045). All efforts were made to minimize the suffering of animals.

### 2.1. Birds, Housing, and Vaccination

A total of 120, 1-day-old, commercial, unsexed Cobb 500 chicks, obtained from Arab Poultry Breeders Co. Limited (Ommat, Egypt), were individually wing-banded, and randomly assigned to four treatment groups (30 per group). Each group was subdivided into three replicates of 10 per each. The experiment was conducted using unsexed chicks and they were randomly distributed. For sampling, 3 samples were collected from each replicate, including 1 male and 2 from females in all groups, to avoid the sex effect. Birds were raised in wired cages and received an experimental diet for five consecutive weeks. The ambient temperature was maintained at 32 °C in the first week, and gradually decreased (3 °C/week) to 21 °C on the 5th week. Chicks were exposed to continuous light during the first two days of age, and then exposed to light for 23 h, followed by an hour of darkness per day thereafter. Diets and fresh water were supplied *ad libitum*. The chicks in all the cages were vaccinated against Newcastle disease virus (NDV), using Hitchner B1 (Izovac^®^ HitcherB1+ H120 Batch no 1717, Brescia, Italy) at 7 days old (DO) and LaSota (Nobilis^®^ ND LaSota, Intervet, Netherlands) at 17 and 27 DO and also vaccinated against infectious bursal disease (IBD) using an intermediate strain vaccine (Bursine 2^®^ vaccine, Zoetis, Parsippany, CA, USA) at 12 and 23 DO. All vaccines were applied using the eye drop method. 

### 2.2. Diets and Experimental Design

The basal diets (BD; corn-soybean-based) for the starter phase (days 1–21) and the finisher phase (days 22–35) met the recommendation of the National Research Council (NRC) [19] for broiler chickens. The commercial diets were chemically analyzed [20], as shown in Table 1. The experimental groups were as follows: G1, BD control group; G2, BD plus *C. butyricum*-based probiotic (Clostri-mix^®^, Cheil Bio Co, Ltd., Youngdungpo-Gu, Seoul, South Korea; every kilogram of Clostri-mix^®^ contained more than 1.0 × 10^10^ CFU *Clostridium butyricum* and excipient) at 0.5 g/kg diet; G3, BD plus *S. cerevisiae*-based probiotic (Top Bio Yeast^®^, Top Bio-Technology Co. Ltd, Tangshan, China; containing 100% active yeast cells) at 0.5 g/kg diet and; G4, BD plus 0.25 g/kg Clostri-mix^®^ plus 0.25 g/kg Top Bio Yeast^®^. To assure the homogenous distribution of additives in final feeds, one common batch (as mentioned in Table 1) was produced, and then divided into 4 sub-batches fortified with additives.

### 2.3. Growth Measurements

The initial body weight (iBW), final body weight (fBW), feed intake (FI), and BWG were recorded, whereas the FCR and protein efficiency ratio (PER) were calculated, according to the methods described by Brody [21] and McDonald et al. [22], respectively. In addition, mortality in chicks was recorded daily throughout the experimental period. Each cage contained one feeder, which hanged outside the cage to decrease feed wastes. The diet was provided *ad libitum* and the daily feed intake was calculated by the difference between the weight of offered feed and the remained part, divided by the number of birds in each cage and totalized to be per week. The PER was calculated by dividing the number of grams of weight gain produced by weight of dietary protein consumed [22], as mentioned in Material and Methods section:

(1) $$\mathrm{PER}=\frac{\mathrm{Body}\;\mathrm{weight}\;\mathrm{gain}\;\left(g\right)/\mathrm{bird}/\mathrm{week}}{\mathrm{Protein}\;\mathrm{intake}\;\left(g\right)/\mathrm{bird}/\mathrm{week}}$$

However, FCR was calculated according to the equation:

(2) $$\mathrm{FCR}=\frac{\mathrm{Feed}\;\mathrm{intake}\;\left(g\right)}{\mathrm{Body}\;\mathrm{weight}\;\mathrm{gain}\;\left(g\right)}$$

### 2.4. Sample Collection 

Blood samples (*n* = 9; 3 chicks from each replicate in each treatment) were collected early in the morning from wing vein and centrifuged (2661× *g* for 5 min at 4 °C) at 21 and 35 days of age to extract serum, which was used for the hemagglutination inhibition (HI) test. The clear sera were obtained from the collected samples for the HI test for the NDV. Additionally, at 21 and 35 days of age, fecal samples (*n* = 9; 3 chicks from each replicate in each treatment) were collected and transferred to buffered peptone water (BPW, HiMedia Laboratories, Mumbai, India), which was immediately used for counting the intestinal microbes. On day 35, nine birds (3 chicks from each replicate in each group) were killed under anesthesia with an intravenous injection of sodium pentobarbital (50 mg/kg) (CAMEO Chemicals, Tampa,FL, USA) and necropsied for carcass traits. Additionally, 3 cm of duodenal samples were collected, washed with saline, and maintained in neutral buffered formalin (10%) (Algomhoria Co. Cairo, Egypt) for histological examination. The fixed samples were processed with the conventional paraffin-embedding technique. Histological sections of 3 μM were prepared from the paraffin blocks of samples. These sections were stained with the Hematoxylin and Eosin (H&E) technique [23].

### 2.5. Intestinal Microbial Counting

#### 2.5.1. Total Coliform Count 

Ten-fold dilutions (10^−1^ to 10^−7^) were performed with BPW for each fecal sample, cultured, and incubated aerobically at 37 °C for 24 h on MacConkey’s agar (Oxoid Laboratories, England, UK). Following incubation, all the red colonies within the range of 15–150 µM were selected for the total coliform count [24].

#### 2.5.2. Total Clostridial Count 

*Clostridium perfringens* colonies were subcultured on Perfringens agar [tryptose sulphite cycloserine (TSC agar), mixed with a selective supplement, 400 mg of D-cycloserine (Oxoid Laboratories, England, UK) per liter in the same previous dilutions, and anaerobically incubated at 37 °C for 48 h using CO_2_ gas generating kits (Oxoid Laboratories, England, UK). Black colonies within the range of 25–250 µM were selected for counting [25].

#### 2.5.3. Total *Lactobacillus* Count

*Lactobacillus* was cultured on Rogosa agar plates (HiMedia Laboratories, Mumbai, India) as per the same previous dilutions and incubated at 37 °C with 5% CO_2_ for 48 h. All whitish colonies that appeared on the plates were counted as *Lactobacillus* [26].

### 2.6. Hemagglutination Inhibition (HI) Test

NDV antigen (LaSota strain) was used to test the chick serum samples collected at 21 and 35 days of age (9 samples, 3 chicks from each replicate in each treatment group) to measure antibody titers against NDV in all the chicken groups using the HI test. HI is an indicator of the immunity of the birds in all the treatment groups. The HI titer was expressed as the reciprocal of the highest dilution that caused inhibition of agglutination, and the geometric mean titer (GMT) was calculated according to the World Organization for Animal Health (OIE) [27].

### 2.7. Carcass Traits

At the end of the experiment, 3 chicks from each replicate in each treatment group (*n* = 9) were randomly selected, weighed, slaughtered, and exsanguinated, and the dressed weight was calculated according to Price [28]. The internal organs and parts of the carcass were expressed relative to live body weight.

### 2.8. Histology

Nine duodenal samples from each experimental group were collected and prepared; quantitatively computerized morphometric analysis was performed on the obtained images from the prepared sections and examined microscopically for histomorphological changes [29]. Images were analyzed using ImageJ software, version 1.48 (the National Institute of Health, Bethesda, MD, USA) to measure the villi height, crypt depth, width of the villi at the crypt/villus junction as well as the tip. The measurement was based on the reported mean value of 15 villi/sample (Magnification power, X10).

### 2.9. Statistical Analysis

The data obtained were subjected to Analysis of Variance (ANOVA) appropriate for a completely randomized design, using the statistical program SPSS.20^®^ (IBM Cooperation, Armonk, NY, USA) to assess the significant differences with Duncan’s multiple range test. The statistical model was:

Y_ij_ = μ + T_i_ + e_ij_ (3)

where Y_ij_ is an observation, μ is the overall mean, T_i_ is the effect of treatments (G1, G2…G4) and e_ijk_ is the random error. The differences among means were determined using the *post-hoc* Newman–Keuls test. Statements of statistical significance were based on *p* < 0.05.

## 3. Results

### 3.1. Growth Measurements and Mortality Rate

The results of the growth performance parameters are shown in Table 2. At the end of the trial, only a numeric increase in fBW in all the probiotic-supplemented groups was observed. The highest fBW was observed in the G4 chicks that were fed BD supplemented with a mixture of *C. butyricum* and *S. cerevisiae* (0.25 g/kg diet for each strain), followed by the G2 and G3 chicks, which showed an increase in fBW of 5.5%, 4.9%, and 2.6%, respectively, compared with controls. 

Moreover, the FCR, BWG and PER were significantly increased (*p* < 0.05) in the G4 chicks; however, these values were not significantly increased in the other probiotic-supplemented groups compared to those of the control group. The mortality percentage was reduced in the probiotic-supplemented groups, particularly following the addition of both types of probiotics (G4), compared to that of the control group.

### 3.2. Histological Study

**Figure 1:**
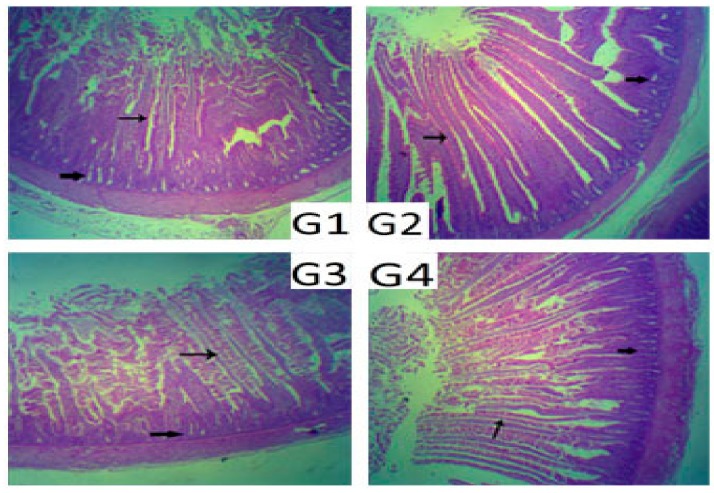
Histological examination of duodenum samples stained with H&E; (**G1**) control (×40), (**G2**) group 2 (×40), (**G3**) group 3 (×40), and (**G4**) group 4 (×40). Small arrows point to intestinal villi. Large arrows point to intestinal crypts.

The histological quantitation of duodenal parameters of probiotic effects, including the villi height, crypt depth, and villi height/crypt depth ratio, are presented in Table 3 and illustrated in Figure 1. Quantitative analysis of villi height and crypt depth showed that the villi height was elongated and the villus height/crypt depth ratio was significantly increased (*p* ˂ 0.05) in the G4 and G2 chicks, in comparison to those of the controls. Moreover, the crypt depth was significantly lower in all the probiotic-supplemented groups than in the control group.

### 3.3. Total Intestinal Bacterial Count

Regarding intestinal microbiota, it was obvious that inclusion of *C. butyricum* preparation at 0.5 g/kg diet (G2) and the mixture of *C. butyricum* preparation and *S. cerevisiae* preparation each at 0.25 g/kg diet (G4) significantly (*p* < 0.05) decreased the total coliform and *Clostridial* counts, in comparison to the use of only *S. cerevisiae* preparation at 0.5 g/kg diet (G3) and the control diet (G1). Moreover, the lowest *Lactobacillus* count was observed at 35 days of age in G1 chicks compared with other groups (Table 4).

### 3.4. Haemagglutination Inhibition (HI) Test

At 35 days of age, HI titers were markedly increased in the G2 and G4 chicks, compared to those in G3 and control chicks (Table 5).

### 3.5. Carcass Traits

Data on the effects of *C. butyricum* and *S. cerevisiae* supplementation on some carcass traits are shown in Table 6. Only the percentages of proventriculus and abdominal fat were significantly affected.

## 4. Discussion

The beneficial effects of individual supplementation with *C. butyricum or S. cerevisiae* probiotics or their mixture on the growth measurements are similar to results by Bostami et al. [30], who compared the growth stimulatory effects among birds fed an antibiotic and birds that were administered a probiotic mixture (comprising different combination of *Bacillus*, *Lactobacillus*, *Saccharomyces*, *Streptococcus*, *Enterococcus*, and *Clostridium*) with feed and water. In addition, these authors included multistrain species probiotics composed of *Bacillus*, *Lactobacillus*, *Saccharomyces*, and *Rhodopseudomonas* in the diet [31], and concluded that the *Rhodopseudomonas*-based probiotic mixture had the ability to be used as an alternative feed additive to antibiotics in broilers. 

The improvement in Bw, BWG, FCR, and PER values in G4 chicks might be attributed to the positive effects of probiotics. The probiotics assisted in stabilizing beneficial gut microbes, increasing the digestion of nutrients, stabilizing beneficial gut microbes, enhancing immuno-modulation, and increasing the intestinal villi length, thereby increasing the surface area of absorption, and improving bird health [12,32]. The growth promoting effect observed in the probiotic mixture group (G4) could be attributed to the synergistic actions of *C. butyricum* and *S. cerevisiae*, which contained mannan oligosaccharides (derived from the cell wall of *S. cerevisiae*; affect through fermentation of different sugars and synthesis of enzymes that can help in better utilization of nutrients and act as a substrate for *C. butyricum* in the gut of broilers) [18,33]. In addition, probiotic bacteria can protect against several harmful pathogens.

The results of the present study showed a reduction in the mortality rate of the probiotic-supplemented chicks; this result is similar to that of Cmiljanic et al. [34] , who reported that mortality percentage could be decreased by the dietary inclusion of Paciflor-C^®^ as *Arbor Acres* broilers. The percentage of decreased mortality might be related to the defensive function of the probiotics on the gut wall, thereby enhancing the immune response [35]. In addition, the *C. butyricum*-based probiotic had more beneficial effects on the performance parameters in comparison to the addition of *S. cerevisiae*, emphasizing the improved results of the probiotic mixture group due to the synergistic action between the two probiotic strains. These effects could be attributed to the fact that *C. butyricum* produces butyric acid that promotes nutrient metabolism and modulates gut microbiota [13]. Moreover, Nakanishi et al. [36] found that *C. butyricum* could produce large amounts of short-chain fatty acids, such as butyrate and acetate, which are important energy resources for animals and stimulate colonic sodium and fluid absorption. 

Kim et al. [4] and Sen et al. [37] found that dietary probiotic supplementation increased the villus height and villus height/crypt depth ratio, but decreased the crypt depth in broilers. These results support our findings in Table 3. In addition, the results of the present study are in agreement with those of Zhang et al. [8], who found that dietary inclusion of *C. butyricum* in diets increased the jejunal villus height and relative length of the cecum in broilers. These results could be attributed to the production of butyric acid by *C. butyricum* that might provide the energy required for epithelial growth [38]. Our results suggest that dietary inclusion of *C. butyricum* in broiler chicks, separately or in combination with *S. cerevisiae*, was beneficial to the intestinal morphology in broiler chickens. The enhancements of villus length and crypt depth in broiler chickens are important in facilitating the absorption of nutrients and improving the efficiency of the gut. This might explain the better growth performance of the G3 and G4 chicks in the present study in comparison to the other treatment groups. 

Results of intestinal microbiota (Table 4) indicated a significant decrease (*p* ≤ 0.05) in both total coliform and clostridial counts in birds of G2 and G4 compared to the other two groups. Regarding *Lactobacillus* counts, the main significant increase (*p* ≤ 0.05) was in birds of G4 as 7.4 log_10_ CFU/g. These results are supported by Zhang et al. [9] who concluded that *C. butyricum* inhibited the growth of harmful microbes, such as *Escherichia coli* (*E. coli*), whereas the growth of beneficial bacteria, such as *Lactobacillus* and *Bifidobacterium*, were enhanced. Moreover, *Clostridium* can produce elevated levels of short-chain fatty acids, which exhibit therapeutic, bactericidal, and anti-inflammatory effects. This helps in increasing intestinal acidity, which suppresses the growth of harmful intestinal microbes, such as *E. coli* [12], and inhibits the production of Shiga-like toxins and secretion of substances, such as bacteriocins, organic acids, and hydrogen peroxides [39]. 

Consistent with the present observation, Gunal et al. [40] found decreased total bacterial counts in broilers in response to probiotic mixture. The reduction of total bacterial count into the ileum and cecum is assumed to be due to the suppression of potentially pathogenic micro-organisms into the gastrointestinal tract of broiler [41]. Competitive exclusion of beneficial micro-organisms prevents attachment of pathogenic bacteria by lowering intestinal pH content, which inhibits the growth of pathogenic micro-organisms in the intestine [39]. Probiotics or Beneficial Microbes (BM) are involved in protection against a variety of pathogens in chickens including *E. coli*, *Campylobacter* and *Salmonella* and can reduce the mortality of birds [42]. *C. butericum* can inhibit the growth of pathogenic bacteria such as *E. coli* and inhibit the production of Shiga-like toxins, improving the growth performances, humoral immune response and reduced mortality [39].

It has been previously discovered that the intestinal immunity plays a major role in bird’s immune response [43]. Here, we measured NDV HI titers as one of the indicators for effective intestinal immune response (gut-associated lymphoid tissues (GALT)) after using a live vaccination for NDV through eye drops and an increase of HI titers in the G2 and G4 chicks compared to those in G3 and control chicks, which are consistent with those reported by Yang et al. [12], who found that dietary inclusion of *C. butyricum* as an alternative to antibiotic/antibacterial agents, such as colistin sulfate, stimulated immune response and modulated the gut microbiota of broilers. Probiotics exert a defensive function on the intestinal wall and immune system [35], which might help to reduce the mortality. Zhang et al. [9] reported that dietary inclusion of *C. butyricum* exhibited similar or better results in alleviating the immune stress in *E. coli* K88-challenged broilers. In addition, *Clostridium* is more effective in stimulating immune response and reducing mortality in chicks, owing to its synergistic and biotherapeutic effects [39].

Regarding carcass traits, similar findings were reported by Rezaeipour et al. [44], who stated that carcass traits were not affected by L-threonine or *S. cerevisiae* supplementations in broilers. However, our results showed that the percentage of abdominal fat was significantly lower (*p* < 0.05) in the G4 group than those of the other groups. In conformity with our findings, Liao et al. [45] showed that supplementing broiler diets with 1  ×  10^9^ CFU of *C. butyricum*/kg decreased the percentage of abdominal fat (*p* ˂ 0.05) compared to that of the controls. These varying results might be attributed to the different doses or types of probiotics used, strain of broilers, BD, or environmental conditions. In partial agreement, Mohamed et al. [46] stated that addition of yeast to broiler diets did not affect the percentage of dressing and relative weight of heart, gizzard, and abdominal fat; however, all the carcass parameters and weights of the internal organs were significantly (*p* ≤ 0.05) affected by the dietary treatments, which were also supported by Paryad and Mahmoudi [47]. Similar to our findings, Liao et al. [45] showed that supplementing broiler diets with 1 × 10^9^ CFU of *C. butyricum*/kg decreased the percentage of abdominal fat (*p* ˂ 0.05) compared to that of the controls. These varying results might be attributed to the different doses or types of probiotics used, strain of broilers, BD, or environmental conditions.

## 5. Conclusions

From the findings of our study, we concluded that the use of probiotics containing both *C. butyricum* and *S. cerevisiae* in the broiler diet was beneficial in enhancing the total BWG and PER, while decreasing the percentage of abdominal fat. Application of the probiotic mixture in a half dose or *C. butyricum* in a full dose increased the villi height, villus height/crypt depth ratio, *Lactobacillus* count, and HI titers for NDV, whereas it decreased the total fecal coliform and *Clostridial* counts. It can be further concluded that the dietary supplementation of a combined mixture of *C. butyricum* and *S. cerevisiae* in an equal ratio is more effective in enhancing the growth performance, immune status, and gut health of broilers, in comparison to individual supplementation at a full dose.

## Figures and Tables

**Table 1:** Ingredients and nutrient composition of the diets (as-fed basis).

Ingredients	Starter Diet	Grower Diet
Yellow corn (CP 8.5%)	54	60.82
Soybean meal (CP 44%)	33.29	26.47
Corn gluten (CP 60%)	6	5
Soybean oil	2.3	3.44
Limestone	1.35	1.53
Dicalcium phosphate	1.74	1.47
L-Lysine ^1^	0.12	0.13
Dl-methionine ^2^	0.5	0.44
Vit. and mineral premix ^3^	0.3	0.3
NaCl	0.3	0.3
Antimold (Mold Tox) ^4^	0.1	0.1
Total	100	100
*Calculated composition* ^5^		
Metabolizable energy (Kcal Kg^-1^)	3020	3165
Lysine %	1.3	1.1
Methionine + Cystine%	0.92	0.8
Na%	0.15	0.15
*Analyzed composition* ^6^		
Crude protein %	23.0	20.0
Calcium %	1.0	0.9
Available phosphorus %	0.46	0.40

^1^ L-lysine, 99% feed grade, ^2^ Dl-methionine, 99% feed grade, China, ^3^ Vitamin and mineral premix (Hero mix) produced by Heropharm and composed (per 3 kg) of vitamin A, 12,000,000 IU; vitamin D3, 2,500,000 IU; vitamin E, 10,000 mg; vitamin K3, 2000 mg; vitamin B1, 1000 mg; vitamin B2, 5000 mg; vitamin B6, 1500 mg; vitamin B12, 10 mg; niacin, 30,000 mg; biotin, 50 mg; folic acid, 1000 mg; pantothenic acid, 10,000 mg; manganese, 60,000 mg; zinc, 50,000 mg; iron, 30,000 mg; copper, 4000 mg; iodine, 300 mg; selenium, 100 mg; and cobalt, 100 mg, ^4^ EL TOBA CO. For premixes and feed, El-Sadat city, Egypt, ^5^ Metabolizable energy (Kcal Kg^−1^), Lysine%, Methionine + Cystine% and Na% were calculated according to National Research Council (NRC) [19], ^6^ Analyzed according to Association of Official Analytical Chemists (AOAC) [20]. CP: Crude protein.

**Table 2:** Effect of dietary supplementation of *Clostridium butyricum* (*C. butyricum*) and *Saccharomyces cerevisiae* (*S. cerevisiae*) on the growth indices of broiler chickens.

Parameter	Groups				*p*-Value
	G1	G2	G3	G4	
Initial body weight (iBW, g)	41 ± 0.45	41.71 ± 0.67	41.84 ± 0.49	42.1 ± 0.43	0.569
Final body weight (fBW, g)	1786.67 ± 40.78	1877.89 ± 38.34	1833.75 ± 48.11	1891.9 ± 30.43	0.514
fBW (RTC *)	100	105.11	102.64	105.89	0.512
Body weight gain (BWG, g)	1714.05 ± 52.56 ^b^	1836.53 ± 38.17 ^ab^	1792.45 ± 47.93 ^ab^	1849.62 ± 30.58 ^a^	0.548
BWG (RTC)	100 ^b^	107.15 ^a^	104.57 ^ab^	107.91 ^a^	0.023
Total feed intake (TFI, g)	3198.88 ± 10.55	3211.69 ± 6.76	3209.17 ± 8.03	3212.81 ± 8.11	0.668
TFI (RTC)	100	100.40	100.32	100.44	0.658
Feed conversion ratio (FCR)	1.91 ± 0.08 ^a^	1.76 ± 0.04 ^a^^b^	1.81 ± 0.05 ^a^^b^	1.75 ± 0.03 ^b^	0.014
FCR (RTC)	100	92.15	94.76	91.62	0.756
Protein efficiency ratio (PER)	2.55 ± 0.06 ^b^	2.66 ± 0.05 ^ab^	2.60 ± 0.07 ^ab^	2.68 ± 0.04 ^a^	0.005
Mortality %	10 ± 5.77 ^b^	6.6 ± 3.33 ^ab^	6.6 ± 6.67 ^ab^	3.3 ± 3.33 ^a^	0.021

G1 = basal diet (BD), G2 = BD plus 0.5 g/kg diet *C. butyricum* preparation, G3 = BD plus 0.5 g/kg diet *S. cerevisiae* preparation, and G4 = BD plus 0.25 g/kg *C. butyricum* preparation plus 0.25 g/kg *S. cerevisiae* preparation. * RTC = Relative to control. ^a,b^ Means within the same row carrying different superscripts are significantly different (*p* ≤ 0.05).

**Table 3:** Effect of dietary supplementation of *C. butyricum* and *S. cerevisiae* on the villi length (µM) and crypt depth (µM) in broiler chickens.

Morphology	Groups				*p* Value
	G1	G2	G3	G4	
Villi height	5.18 ± 0.13 ^b^	7.660 ± 0.17 ^a^	5.435 ± 0.93 ^b^	7.862 ± 0.12 ^a^	0.004
Crypt depth	1.01 ± 0.34 ^a^	0.649 ± 0.03 ^b^	0.620 ± 0.01 ^b^	0.654 ± 0.05 ^b^	0.012
Villus height/crypt depth ratio	5.13 ± 0.11 ^b^	11.80 ± 0.04 ^a^	8.77 ± 0.01 ^ab^	12.02 ± 0.07 ^a^	0.001

G1 = basal diet (BD), G2 = BD plus 0.5 g/kg diet *C. butyricum* preparation, G3 = BD plus 0.5 g/kg diet *S. cerevisiae* preparation, and G4 = BD plus 0.25 g/kg *C. butyricum* preparation plus 0.25 g/kg *S. cerevisiae* preparation. ^a,b^ Means within the same row carrying different superscripts are significantly different (*p* ≤ 0.05).

**Table 4:** Mean fecal counts of coliform, *Clostridium*, and *Lactobacillus* at 35 days of age in broiler chickens.

Item	Groups				*p* Value
	G1	G2	G3	G4	
*Coliform*	8.34 ± 0.52 ^a^	7.65 ± 0.35 ^b^	7.94 ± 0.41 ^a^	7.60 ± 0.46 ^b^	0.024
*Clostridia*	9.47 ± 0.46 ^a^	9.09 ± 0.29 ^b^	9.30 ± 0.35 ^a^	8.95 ± 0.40 ^b^	0.024
*Lactobacillus*	4.00 ± 0.35 ^b^	6.74 ± 0.46 ^a^	6.69 ± 0.29 ^a^	7.47 ± 0.40 ^a^	0.028

G1 = basal diet (BD), G2 = BD plus 0.5 g/kg diet *C. butyricum* preparation, G3 = BD plus 0.5 g/kg diet *S. cerevisiae* preparation, and G4 = BD plus 0.25 g/kg *C. butyricum* preparation plus 0.25 g/kg *S. cerevisiae* preparation. ^a,b^ Means within the same row carrying different superscripts are significantly different (*p* ≤ 0.05).

**Table 5:** Haemagglutination inhibition (HI) titers for Newcastle disease virus (NDV) in the collected serum samples at 21 and 35 days of age in broiler chickens.

Item	Groups				*p* Value
	G1	G2	G3	G4	
*Coliform*	4.20 ± 0.29	4.30 ± 0.21	4.10 ± 0.28	4.00 ± 0.21	0.062
*Clostridia*	6.10 ± 0.35 ^a^	7.00 ± 0.15 ^b^	6.00 ± 0.37 ^a^	7.00 ± 0.15 ^b^	0.045

G1 =basal diet (BD), G2 = BD plus 0.5 g/kg diet *C. butyricum* preparation, G3 = BD plus 0.5 g/kg diet *S. cerevisiae* preparation, and G4 = BD plus 0.25 g/kg *C. butyricum* preparation plus 0.25 g/kg *S. cerevisiae* preparation. ^a,b^ Means within the same row carrying different superscripts are significantly different (*p* ≤ 0.05).

**Table 6:** Effect of dietary supplementation of *C. butyricum* and *S. cerevisiae* on some carcass traits in broiler chickens.

Variables (% of Slaughter Weight)	Groups				*p* Value
	G1	G2	G3	G4	
Dressing	72.51 ± 1.49	72.79 ± 0.95	71.26 ± 1.63	71.54 ± 1.32	0.544
Liver	2.65 ± 0.16	2.24 ± 0.38	2.91 ± 0.08	2.37 ± 0.17	0.568
Heart	0.56 ± 0.02	0.53 ± 0.05	0.43 ± 0.01	0.46 ± 0.02	0.875
Spleen	0.11 ± 0.01	0.11 ± 0.01	0.09 ± 0.01	0.10 ± 0.01	0.652
Proventriculus	0.47 ± 0.07 ^ab^	0.44 ± 0.02 ^b^	0.51 ± 0.01 ^ab^	0.61 ± 0.05 ^a^	0.012
Gizzard	1.61 ± 0.04	1.46 ± 0.05	1.47 ± 0.08	1.55 ± 0.17	0.452
Abdominal fat	1.32 ± 0.07 ^a^	1.03 ± 0.27 ^ab^	0.97 ± 0.16 ^ab^	0.74 ± 0.05 ^b^	0.025
Thymus gland	0.35 ± 0.03	0.36 ± 0.04	0.29 ± 0.05	0.41 ± 0.06	0.365
Bursa	0.04 ± 0.03	0.06 ± 0.01	0.07 ± 0.03	0.07 ± 0.03	0.875
Intestine	5.86 ± 0.28	5.73 ± 0.18	5.59 ± 0.22	5.67 ± 0.19	0.245
Intestine Length (cm)	9.85 ± 0.18	9.81 ± 0.28	9.97 ± 0.38	10.22 ± 0.10	0.651

G1 = basal diet (BD), G2 = BD plus 0.5 g/kg diet *C. butyricum* preparation, G3 = BD plus 0.5 g/kg diet *S. cerevisiae* preparation, and G4 = BD plus 0.25 g/kg *C. butyricum* preparation plus 0.25 g/kg *S. cerevisiae* preparation. ^a,b^ Means within the same row carrying different superscripts are significantly different (*p* ≤ 0.05).

## Abbreviations

NDV	Newcastle disease virus
EU	European Union
BD	basal diet
iBW	initial body weight
fBW	final body weight
FI	feed intake
BWG	body weight gain
FCR	feed conversion ratio
PER	protein efficiency ratio
HI	hemagglutination inhibition
TSC	tryptose sulphite cycloserine
GMT	geometric mean titer
ANOVA	analysis of variance
